# Unitary Transformations in the Quantum Model for Conceptual Conjunctions and Its Application to Data Representation

**DOI:** 10.3389/fpsyg.2015.01734

**Published:** 2015-11-12

**Authors:** Tomas Veloz, Sylvie Desjardins

**Affiliations:** ^1^Department of Mathematics, University of British ColumbiaKelowna, BC, Canada; ^2^Center Leo Apostel, Vrije Universiteit BrusselBrussels, Belgium; ^3^Instituto de Filosofía y Ciencias de la Complejidad - IFICCÑuñoa, Chile

**Keywords:** concept combination, quantum cognition, data representation, unitary transformation, conjunction

## Abstract

Quantum models of concept combinations have been successful in representing various experimental situations that cannot be accommodated by traditional models based on classical probability or fuzzy set theory. In many cases, the focus has been on producing a representation that fits experimental results to validate quantum models. However, these representations are not always consistent with the cognitive modeling principles. Moreover, some important issues related to the representation of concepts such as the dimensionality of the realization space, the uniqueness of solutions, and the compatibility of measurements, have been overlooked. In this paper, we provide a dimensional analysis of the realization space for the two-sector Fock space model for conjunction of concepts focusing on the first and second sectors separately. We then introduce various representation of concepts that arise from the use of unitary operators in the realization space. In these concrete representations, a pair of concepts and their combination are modeled by a single conceptual state, and by a collection of exemplar-dependent operators. Therefore, they are consistent with cognitive modeling principles. This framework not only provides a uniform approach to model an entire data set, but, because all measurement operators are expressed in the same basis, allows us to address the question of compatibility of measurements. In particular, we present evidence that it may be possible to predict non-commutative effects from partial measurements of conceptual combinations.

## 1. Introduction

### 1.1. Concept combinations in quantum cognition

The application of quantum models to cognitive phenomena is an emergent field known as quantum cognition (Aerts, 2009; Pothos and Busemeyer, 2013). One of the areas in quantum cognition that has received much attention is the study of concepts and their combinations (Aerts and Gabora, 2005a,b; Aerts, 2007a,b; Aerts and Sozzo, 2011; Aerts et al., 2013). In a general setting, a cognitive situation might include multiple concepts forming aggregated structures (Rips, 1995; Fodor, 1998). For example, the concepts “*Fruit*” and “*Vegetable*” can be combined to form a new concept “*Fruit And Vegetable*” (Hampton, 1988a). This example of a concept combination is built with the connective “And,” which is also an operation mathematically defined in logic and probability. The question becomes, is it possible to apply the mathematical definition of the connective “*And*” to build the structure of “*Fruit And Vegetable*” from the structures of “*Fruit*” and “*Vegetable*”? Cognitive scientists have performed several experiments measuring various semantic estimations including typicality, membership, and similarity of concept combinations built with connectives such as “ *And*,” and “*Not*” (Hampton, 1997a, 1988a,b), and adjective-noun compounds such as “*Red Apple*” (Medin and Shoben, 1988; Medin, 1989; Kamp and Partee, 1995). The evidence collected during two decades of research suggests that it might not be possible to represent all the experimental data for concept combinations using the mathematical structures of fuzzy logic or probability theory. Quantum cognition proposes an alternative approach.

While traditional models based on classical logic, probability, or fuzzy set theory have failed to properly account for cognitive phenomena exhibiting non-classical probabilistic features, quantum models have consistently provided a framework that easily encompasses these and other so-called cognitive biases (Gilovich et al., 2002; Busemeyer et al., 2011) or paradoxical phenomena (Aerts et al., 2011a). Quantum inspired models have been successfully developed in the areas of decision making (Aerts et al., 2011b, 2012b; Busemeyer et al., 2011; Busemeyer and Bruza, 2012), psychology of categorization (Aerts and Aerts, 1995; Blutner et al., 2013; Sozzo, 2014), human memory (Bruza and Cole, 2005; Bruza et al., 2009, 2012), and finances (Khrennikov, 2009; Haven and Khrennikov, 2013). In this paper we will focus on the phenomena of concept conjunction. However, since our analysis and methodology is based on pure mathematical notions of the quantum mechanical framework, the results presented in this paper can be extended to other concept combinations (Veloz, 2015).

Aerts (2009) formally states the conditions that characterize the existence of a classical probability model for concept conjunction:

Definition 1. *Let μ(A), μ(B), and μ(AB) be the membership weights of an exemplar p with respect to a pair of concepts* A *and* B *and their conjunction* AB. *We say that these membership weights are classical conjunction data if there exists a Kolmogorovian probability space* (Ω, σ(Ω), *P*), *and events E_A_, E_B_* ∈ σ(Ω) *such that*

(1) $$\begin{array}{l} P(E_{A})=\mu (A),\\ P(E_{B})=\mu (B),\\ P(E_{A}\cap E_{B})=\mu (AB). \end{array}$$

Classical conjunction data characterizes the membership values of the conjunction of concepts that can be modeled in a classical probabilistic framework. It is therefore important to characterize the notion of classical conjunction data in terms of the membership weights.

Corollary 1. *The membership weights μ(A), μ(B), and μ(AB) of an exemplar p with respect to concepts* A, B, *and their conjunction* AB *are classical conjunction data if and only if*

(2) 0≤μ(AB)≤μ(A),

(3) 0≤μ(AB)≤μ(B),

(4) 0≤μ(A)+μ(B)−μ(AB)≤1.

A large body of experimental evidence and a considerable amount of data analysis indicate that the membership of exemplars with respect to concept combinations does not form classical conjunction data (Fodor and Lepore, 1996; Hampton, 1997a,b; Aerts and Gabora, 2005a,b). Namely, the membership with respect to the conjunction of concepts is generally larger than the membership of one of the former concepts, and thus violates either conditions (2) or (3). This phenomenon is called single overextension. When conditions (2) and (3) are violated simultaneously, it is called double overextension. The violation of condition (4) is called the Kolmogorovian factor violation. We refer to (Pitowsky, 1989; Aerts, 2009) for an explanation of this phenomenon.

In Supplementary Table 1, we show two cases reported in Hampton (1988b). In the first case, the membership weight μ_1_(*AB*) of the item *p*_1_ = “coffee table” with respect to the conjunction A_1_B_1_ = “*Furniture And Household Appliances*” is single overextended with respect to the membership weights μ_1_(*A*) and μ_1_(*B*) of concepts A_1_ = “*Furniture*,” and B_1_ = “*Household Appliances*,” respectively. In the second case, membership weight μ_2_(*AB*) of the item *p*_2_ = “tree house” with respect to the conjunction A_2_B_2_ = “*Building And Dwelling*” is doubly overextended with respect to the membership weigths μ_2_(*A*) and μ_2_(*B*) of the concepts A_2_ = “*Building*,” and B_2_ = “*Dwelling*,” respectively.

The phenomenon of overextension has also been demonstrated not only for membership estimations, but also in typicality (Smith and Osherson, 1981; Hampton, 1996; Storms et al., 1998), property relevance (Fodor and Lepore, 1996; Hampton, 1997a,b; Aerts and Gabora, 2005a,b), and probability estimations (Tversky and Kahneman, 1983; Moro, 2009).

### 1.2. The quantum approach to concept combination

The quantum approach to concepts introduces two fundamental assumptions that depart from classical approaches:

**Table:** 

**A1**	Concepts are not represented by a set of instances. Instead, a concept is assumed to exist in a state. A Hilbert space H is introduced, and a unit vector |ψ〉 ∈ H represents the state of the concept.
**A2**	Semantic estimations are not functions over the set of instances. Instead, a semantic estimation is a measurement operator, **M** : H→H, that projects onto a subspace of H.

Concepts A and B are represented by the states |*A*〉 and |*B*〉, respectively. When we consider the conjunction AB of these two concepts, there are two different ways to combine the concepts (Aerts, 2009). The first considers the conjunction of concepts from an intuitive perspective in the sense that the connective *And* does not play a logical role in the combination AB; instead the conjunction AB is viewed as an emergent entity. In particular, the quantum model assumes that the state of the combined concept |*AB*〉 ∈ H is given by a superposition of the states of concepts A and B as follows:

(5) $$|AB\rangle =\frac{1}{\sqrt{2}}(|A\rangle +|B\rangle ).$$

The second way considers the conjunction of concepts from a logical perspective, in the sense that *And* does play a logical role in the combination AB. In particular, the quantum model assumes that the state of the combined concept |*C*〉 is modeled in the tensor product space H ⊗ H, where each space in the product captures the representation of the concepts in the combination, while the entire space represents the conjunction. The two quantum models of concept combination are presented in Supplementary Material. These two modes can be unified in a mathematical framework developed in quantum mechanics called Fock space (Aerts, 2007a, 2009).

A Fock space is a direct sum of tensor products of Hilbert spaces, where each space in the sum represents the state space of a system having different numbers of particles (Meyer, 1995). For the case of concepts, we model the state of the combination of two concepts in the two-sector Fock space:

(6) F=H⊕(H⊗H).

The first space, H, also called the first sector, represents the concept combination as an emergent entity. The second space, H ⊗ H, called the second sector, represents the concept combination as a logical entity. The state of the combined concept in the two-sector Fock space is hence a superposition of the two modes of combination.

For example, when |*C*〉 = |*A*〉 ⊗ |*B*〉, the state |ψ〉 of the concept combination is 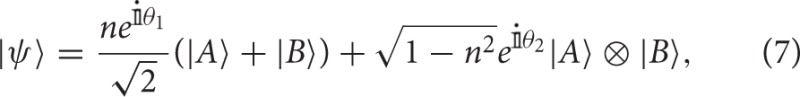 and the membership formula is given by

(8) $$\mu (AB)=n^{2}\left(\frac{\mu (A)+\mu (B)}{2}+ℜ(\langle A|M|B\rangle )\right)+\sqrt{1-n^{2}}\mu (A)\mu (B),$$

for 0 ≤ *n* ≤ 1.

When *n* = 1, the membership weight μ(*AB*) corresponds to the sum of the average of μ(*A*) and μ(*B*), plus an interference term ℜ(〈*A*|*M*|*B*〉) bounded by

$$-\sqrt{\mu (A)\mu (B)}\leq ℜ(\langle A|M|B\rangle )\leq \sqrt{\mu (A)\mu (B)}.$$

In the absence of interference, i.e., when ℜ(〈*A*|*M*|*B*〉) = 0, the membership weight is simply the average of the former membership weights. This particular case, which has been shown to provide a good first approximation to exemplars of conceptual conjunction (Aerts et al., 2012a), is overextended, and therefore non-classical. When *n* = 0, the membership weight corresponds to the product μ(*A*)μ(*B*), which is equivalent to the probability of two joint classical events that are independent. When 0 < *n* < 1, the state of the concept is in the superposition of the two modes of combination.

Finally, the membership operator for a certain exemplar with respect to the conjunction of two concepts is given by

(9) $$\;M^{F}=\text{M}⊕(\text{M}⊕\text{M}),$$

where **M** is the operator that measures membership of the exemplar in the first sector, and **M** ⊗ **M** measures the membership of the exemplar with respect to the two concepts simultaneously in the second sector.

In addition to providing a suitable mathematical framework for cognitive models, quantum cognition also offers a different perspective on cognitive phenomena: uncertainty is described by means of superposed states (Aerts et al., 2011b), non-logical coherence involves interference (Aerts, 2009), order effects are revealed by incompatible measurements (Wang and Busemeyer, 2013), and certain “verb-noun” conceptual combinations mimic the structure of physically entangled particles (Aerts and Sozzo, 2014).

### 1.3. The representation of data

One of the reasons why quantum models of concept combinations have not been widely used is that the issue of data representation has been overlooked. Scholars have studied the capacity of quantum models to fit semantic estimations of concept combinations, and have presented concrete representations of the different estimations to validate the models (Aerts, 2007a,b, 2009; Aerts et al., 2012a; Sozzo, 2014); these concrete representations, however, model the data in an exemplar-based fashion, where one operator is used for all exemplars, but the conceptual state varies with exemplars.

For example, Aerts (2009) builds a quantum model in the Hilbert space ℂ^3^ to consider the exemplars “filing cabinet” and “heated waterbed” with respect to concepts A = “*Furniture*, B = “*Household Appliances*,” and their conjunction AB. For the first exemplar, we have μ(*A*) = 0.97, μ(*B*) = 0.31, and μ(*AB*) = 0.53. This case is represented by the vectors 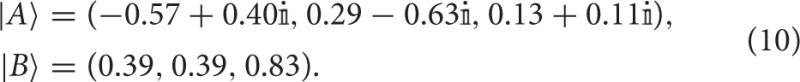 For the second exemplar, μ(*A*) = 1, μ(*B*) = 0.49, and μ(*AB*) = 0.78, and the state vectors are given by

(11) $$\begin{array}{l} |A\rangle =(0.71,0.71,0),\\ |B\rangle =(0.49,0.49,0.71). \end{array}$$

In both cases **M** is defined by the projection operator

(12) M(x,y,z)→(x,0,0).

Such concrete representations are useful to validate models, but unwieldy if one seeks to build a model that can be used for studying and comparing large amounts of data. Because the state is independent of the exemplar, it must remain the same for all measurements. But if we require the state representing the concept to remain fixed, then the number of measurement operators is restricted by the dimension of the Hilbert space H. In fact, because the membership operator is usually represented by the identity projector restricted to a smaller subspace, and the identity operator of the entire space and the null operator entail trivial measurements, the number of projectors available to represent membership measurements is restricted to *n* − 1, for *n* = dim(H). This implies that, if we consider *n* or more exemplars, then some exemplars will not have a unique membership operator. These issues become crucial in real-world situations involving concepts that entail thousands of exemplars (Tenenbaum et al., 2011).

In Section 2, we take a close look at the concrete representations of quantum models on each sector of the Fock space to identify the minimal dimensionality required to reach the modeling capacity of each of the sectors. In Section 3, we introduce the notion of unitary transformation for the first and second sectors of the Fock space separately, and propose concrete representations for concepts in these two models that require a single conceptual state, and a collection of exemplar-dependent operators. In Section 4, we use these representations to advance a conjecture concerning compatibility of measurements.

## 2. Dimensionality analysis of the two-sector Fock space model

In what follows we determine the dimension of H required to model concept combinations in the first and second sectors of the two-sector Fock space model. To explore this question, we assume H = ℂ^*n*^ equipped with the standard inner product, and analyze how *n* relates to the representation of concepts.

### 2.1. First sector dimension analysis

The Hilbert space model for concept conjunction requires two vectors, |*A*〉, |*B*〉 ∈ H, and an orthogonal projector, **M** : H → H, such that

(13) 〈A|A〉=〈B|B〉=1,

(14) 〈A|B〉=0,

(15) 〈A|M|A〉=μ(A), 〈B|M|B〉=μ(B),

(16) $$\mu (AB)=\frac{1}{2}(\mu (A)+\mu (B))+ℜ(\langle A|\text{M}|B\rangle ).$$

The next theorem shows that *n* = 3 is sufficient to build a model that satisfies conditions (13–16).

Theorem 1. *Let μ(A), μ(B), and μ(AB) denote the membership of an exemplar with respect to concepts* A, B, *and their conjunction* AB. *The membership weights are compatible with a complex Hilbert space model* H = ℂ^3^ *if and only if*

(17) μ(AB)∈[ave(AB)−dev(AB),ave(AB)+dev(AB)],

*where*

(18) $$\begin{array}{l} \text{ave}(AB)=\frac{1}{2}(\mu (A)+\mu (B)),\;and\\ \text{dev}(AB)=\sqrt{\min(\mu (A)\mu (B),(1-\mu (A))(1-\mu (B))}. \end{array}$$

*Proof*. We derive Equation (17) by applying conditions (13–16). First, if **M** is a zero- or three-dimensional projector, then

(19) $$\begin{array}{l} \mu (A)=\mu (B)=\mu (AB)=0,\text{ or}\\ \mu (A)=\mu (B)=\mu (AB)=1, \end{array}$$

respectively. Thus, Equation (17) holds, and Equations (13–16) are satisfied by choosing |*A*〉 and |*B*〉 to be any two mutually orthogonal unit vectors.

Next, we consider the cases where **M** is either a one- or two-dimensional projector. We apply conditions (13–16) to vectors |*A*〉 and |*B*〉 in these two cases separately, and combine the results to obtain (Equation 17).

If **M** is a one-dimensional projector, then without loss of generality, we can choose

(20) $$\begin{array}{l} \text{M}(x,y,z)\to (x,0,0),\text{ and}\\ |A\rangle =(a_{1}e^{i\alpha_{1}},a_{2}e^{i\alpha_{2}},a_{3}e^{i\alpha_{3}}),\\ |B\rangle =(b_{1}e^{i\beta_{1}},b_{2}e^{i\beta_{2}},b_{3}e^{i\beta_{3}}). \end{array}$$

Note that conditions (13) and (15) are satisfied by choosing the coefficients in |*A*〉 and |*B*〉 as follows:

(21) $$\begin{array}{l} a_{1}=\sqrt{\mu (A)};\;a_{2}=\sqrt{\lambda }\sqrt{1-\mu (A)}\;;a_{3}=\sqrt{1-\lambda }\sqrt{1-\mu (A)},\\ b_{1}=\sqrt{\mu (B)};\;b_{2}=\sqrt{\kappa }\sqrt{1-\mu (B)}\;;b_{3}=\sqrt{1-\kappa }\sqrt{1-\mu (B)}, \end{array}$$

with 0 ≤ λ ≤ 1, and 0 ≤ κ ≤ 1. Moreover, Equation (16) implies that μ(*AB*) is given by

(22) $$\mu (AB)=\frac{1}{2}(\mu (A)+\mu (B))+\sqrt{\mu (A)\mu (B)}\cos(\alpha_{1}-\beta_{1}).$$

We then apply condition (14) to obtain

(23) $$\begin{array}{l} -\sqrt{\mu (A)\mu (B)}\cos(\gamma_{1})\\ =\sqrt{\left(1-\mu (A))(1-\mu (B)\right)}F(\lambda ,\kappa ,\cos(\gamma_{2}),\cos(\gamma_{3})), \end{array}$$

(24) $$\begin{array}{l} -\sqrt{\mu (A)\mu (B)}\sin(\gamma_{1})\\ =\sqrt{\left(1-\mu (A))(1-\mu (B)\right)}F(\lambda ,\kappa ,\sin(\gamma_{2}),\sin(\gamma_{3})), \end{array}$$

where

(25) $$F(\lambda ,\kappa ,f(x),f(y))\;=\;\left(\sqrt{\lambda \kappa }f(x)\;+\;\sqrt{\left(1-\lambda )(1-\kappa \right)}f(y)\right).$$

Since *F*(λ, κ, cos(γ_2_), cos(γ_3_)) is a convex combination of $\sqrt{\lambda \kappa }$ and $\sqrt{\left(1-\lambda \right)\left(1-\kappa \right)}$, we have

(26) $$|F(\lambda ,\kappa ,\cos(\gamma_{2}),\cos(\gamma_{3}))|\;\leq \;|\sqrt{\lambda \kappa }|+|\sqrt{\left(1-\lambda )(1-\kappa \right)}|.$$

We set

(27) $$\sqrt{\lambda }=\cos(\theta_{1}),\;\sqrt{\kappa }=\cos(\theta_{2}),$$

for θ_1_, θ_2_ in $\left[0,\frac{\pi }{2}\right]$. Then

(28) $$\begin{array}{l} \sqrt{1-\lambda }=\sin(\theta_{1}),\\ \sqrt{1-\kappa }=\sin(\theta_{2}). \end{array}$$

Substituting Equations (27) and (28) in Equation (26), we obtain

(29) $$|F(\lambda ,\kappa ,\cos(\gamma_{2}),\cos(\gamma_{3}))|\;\leq \;|\cos(\theta_{1}-\theta_{2})|\;\leq 1.$$

Then Equation (23) implies that

(30) $$|\sqrt{\mu (A)\mu (B)}\cos(\gamma_{1})|\;\leq \sqrt{\left(1-\mu (A))(1-\mu (B)\right)}.$$

Therefore, the interference term is bounded as follows:

(31) $$\begin{array}{l} |\sqrt{\mu (A)\mu (B)}\cos(\gamma_{1})|\;\leq \min(\sqrt{\mu (A)\mu (B)},\\ \sqrt{\left(1-\mu (A))(1-\mu (B)\right)})\\ =\text{ dev}(AB). \end{array}$$

Next, combining Equations (23) and (24), we obtain

(32) $$\mu (A)\mu (B)=(1-\mu (A))(1-\mu (B))\hat{F}(\lambda ,\kappa ,\gamma_{2},\gamma_{3}),$$

where

(33) $$\begin{array}{l} \hat{F}(\lambda ,\kappa ,\gamma_{2},\gamma_{3})=F^{2}(\lambda ,\kappa ,\cos(\gamma_{2}),\cos(\gamma_{3}))\\ \;+F^{2}(\lambda ,\kappa ,\sin(\gamma_{2}),\sin(\gamma_{3})). \end{array}$$

Hence,

(34) $$\mu (A)+\mu (B)=1+\mu (A)\mu (B)\left(1-\frac{1}{\hat{F}(\lambda ,\kappa ,\gamma_{2},\gamma_{3})}\right).$$

We use the parametrization for λ and κ given by Equation (27), and apply Equations (29–33), to obtain

(35) $$0\leq \hat{F}(\lambda ,\kappa ,\gamma_{2},\gamma_{3})\leq \cos{\left(\theta_{1}-\theta_{2}\right)}^{2}+\sin{\left(\theta_{1}-\theta_{2}\right)}^{2}=1.$$

Combining Equations (35) and (34) yields

(36) μ(A)+μ(B)≤1.

Therefore, when **M** is a one-dimensional projector, conditions (13–16) imply

(37) $$\begin{array}{l} \mu (AB)\in [\text{ave}(AB)-\text{dev}(AB),\text{ave}(AB)+\text{dev}(AB)],\text{ and}\\ \mu (A)+\mu (B)\leq 1. \end{array}$$

Next, consider the case in which **M** is a two dimensional projector. Without loss of generality, we can assume

M(x,y,z)→(x,y,0).

The requirements Equations (13) and (15) are satisfied by choosing the coefficients in |*A*〉, |*B*〉 as follows

(38) $$\begin{array}{l} a_{1}=\sqrt{\lambda }\sqrt{\mu (A)};\;a_{2}=\sqrt{1-\lambda }\sqrt{\mu (A)}\;;a_{3}=\sqrt{1-\mu (A)},\\ b_{1}=\sqrt{\kappa }\sqrt{\mu (B)};\;b_{2}=\sqrt{1-\kappa }\sqrt{\mu (B)}\;;b_{3}=\sqrt{1-\mu (B)}, \end{array}$$

with 0 ≤ λ ≤ 1, and 0 ≤ κ ≤ 1. Moreover, Equation (16) implies that μ(*AB*) is given by

(39) $$\mu (AB)=\frac{1}{2}(\mu (A)+\mu (B))+\sqrt{\mu (A)\mu (B)}F(\lambda ,\kappa ,\cos(\gamma_{1}),\cos(\gamma_{2})).$$

We apply condition (14) to obtain

(40) $$\begin{array}{l} \sqrt{\mu (A)\mu (B)}F(\lambda ,\kappa ,\cos(\gamma_{1}),\cos(\gamma_{2}))\\ =-\sqrt{\left(1-\mu (A))(1-\mu (B)\right)}\cos(\gamma_{3}). \end{array}$$

Since *F*(λ, κ, cos(γ_1_), cos(γ_2_)) ≤ 1, Equation (40) implies that

(41) $$\begin{array}{l} |\sqrt{\mu (A)\mu (B)}F(\lambda ,\kappa ,\cos(\gamma_{1}),\cos(\gamma_{2}))|\;\leq \min(\sqrt{\mu (A)\mu (B)},\\ \;\sqrt{\left(1-\mu (A))(1-\mu (B)\right)})\\ \;=\text{dev}(AB). \end{array}$$

We repeat the procedure used in the one-dimensional case to obtain

(42) $$\mu (A)\mu (B)\hat{F}(\lambda ,\kappa ,\gamma_{1},\gamma_{2})=(1-\mu (A))(1-\mu (B)).$$

Since $0\leq \hat{F}\left(\lambda ,\kappa ,\gamma_{1},\gamma_{2}\right)\leq 1$, Equation (42) yields

(43) 1≤μ(A)+μ(B).

Therefore, when **M** is a two-dimensional projector, conditions (13–16) imply

(44) $$\begin{array}{l} \mu (AB)\in [\text{ave}(AB)-\text{dev}(AB),\text{ave}(AB)+\text{dev}(AB)],\text{ and}\\ 1\leq \mu (A)+\mu (B). \end{array}$$

We complete the proof by merging Equations (37) and (44).

The general case, H = ℂ^*n*^ for *n* > 3, doesn't provide additional modeling power since the condition given by Equation (17) remains. Also, the case H = ℂ^2^ is more restrictive than the H = ℂ^3^ case. In fact, membership data compatible with conditions (13–16) for H = ℂ^2^ must satisfy μ(*A*) + μ(*B*) = 1 (Veloz, 2015).

### 2.2. Second sector dimension analysis

The second sector of the two-sector Fock space requires a concept combination state |*C*〉 ∈ ℂ^*n*^ ⊗ ℂ^*n*^ and an operator **M** : ℂ^*n*^ → ℂ^*n*^, such that |*C*〉 restricted to the first sector represents the concept A, and |*C*〉 restricted to the second sector represents the concept B. However, |*C*〉 cannot in general be decomposed as a tensor product of the type |*C*_*A*_〉 ⊗ |*C*_*B*_〉, for $|C_{A}\rangle ,|C_{B}\rangle \in \;{\mathbb{C}}^{n}$. Therefore, |*C*〉 is usually a non-separable state.

To recover the probabilistic structure of the former concepts in the combination, the operators **M** ⊗ **1** and **1** ⊗ **M** are applied to |*C*〉 to obtain μ(*A*) and μ(*B*), respectively. Moreover, since |*C*〉 as a whole represents the concept combination AB, then the operator **M** ⊗ **M** is applied to |*C*〉 to obtain μ(*AB*).

The following definition summarizes how data is represented in the second sector.

Definition 2. *Let μ = {μ(A), μ(B), μ(AB)} be a triplet denoting the membership of concepts* A, B, *and their conjunction* AB. *We say that the triplet μ admits a representation in* ℂ^*n*^ ⊗ ℂ^*n*^ *if there exists a unit vector* |*C*〉 ∈ ℂ^*n*^ ⊗ ℂ^*n*^, *and an operator* **M**:ℂ^*n*^ → ℂ^*n*^ *such that*

(45) $$\langle C|M^{A}|C\rangle =\langle C|M⊗1|C\rangle =\mu (A),$$

(46) $$\langle C|M^{B}|C\rangle =\langle C|1⊗M|C\rangle =\mu (B),$$

(47) $$\langle C|M^{\wedge }|C\rangle =\langle C|M⊗\text{M}|C\rangle =\mu (AB).$$

Let ${\left\{|i\rangle \right\}}_{i=1}^{n}$ be the canonical basis of ℂ^*n*^. Without loss of generality, we can take **M** to be an orthogonal projector on the subspace of ℂ^*n*^ spanned by the basis elements |1〉, …, |*r*〉, with *r* < *n*. Hence,

$$M(x_{1},\ldots ,x_{n})\to (x_{1},\ldots ,x_{r},0,\ldots ,0).$$

Next, let |*C*〉 be a unit vector in ℂ^*n*^ ⊗ ℂ^*n*^. That is, 
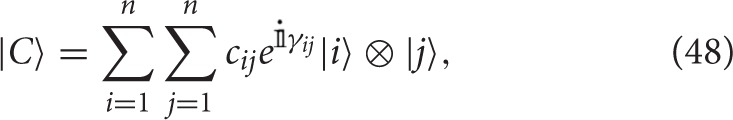
 and 
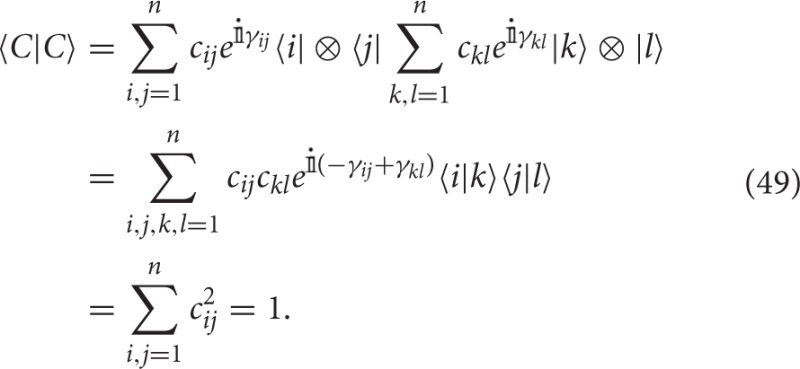
 We now prove that the operator **M** and the vector |*C*〉 above satisfy Equations (45–47) if and only if μ(*A*), μ(*B*), and μ(*AB*) are classical conjunction data.

Theorem 2. *Let μ =* {*μ(A), μ(B), μ(AB)*} *be a triplet denoting the membership of concepts* A, B, *and their conjunction* AB. *The triplet μ is classical conjunction data if and only if it admits a representation in* ℂ^n^ ⊗ ℂ^*n*^ *with n* = 2.

*Proof*. If μ admits a representation in ℂ^2^ ⊗ ℂ^2^, there exists a unit vector |*C*〉 ∈ ℂ^2^ ⊗ ℂ^2^ and an operator **M** such that Equations (45–47) are satisfied. If μ(*A*) = μ(*B*) = μ(*AB*) = 0 or 1, we can choose |*C*〉 to be any unit vector in ℂ^2^ ⊗ ℂ^2^, and **M** to be a zero- or two-dimensional projector, respectively. Otherwise, let {|1〉, |2〉} be the canonical basis for ℂ^2^. Without loss of generality, we can define |*C*〉 by 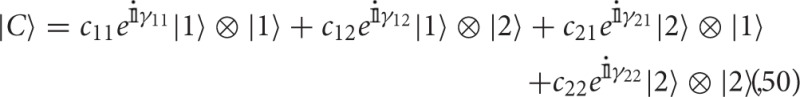 and **M** by the one-dimensional projector into the subspace determined by |1〉. Note that

(51) $$\begin{array}{l} \mu (A)=\langle C|\text{M}⊗1|C\rangle =c_{11}^{2}+c_{12}^{2},\\ \mu (B)=\langle C|1⊗\text{M}|C\rangle =c_{11}^{2}+c_{21}^{2},\\ \mu (AB)=\langle C|\text{M}⊗\text{M}|C\rangle =c_{11}^{2}. \end{array}$$

Then, clearly μ(*AB*) ≤ μ(*A*), μ(*AB*) ≤ μ(*B*), and since |*C*〉 is a unit vector,

(52) $$\mu (A)+\mu (B)-\mu (AB)=c_{11}^{2}+c_{12}^{2}+c_{21}^{2}\leq 1.$$

Therefore, μ is classical conjunction data. The other implication is proven by taking **M** to be the same one-dimensional projector, and |*C*〉 such that

$$\begin{array}{l} c_{11}=\sqrt{\mu (AB)},\\ c_{12}=\sqrt{\mu (A)-\mu (AB)},\\ c_{21}=\sqrt{\mu (B)-\mu (AB)},\\ c_{22}=\sqrt{1-\mu (A)-\mu (B)+\mu (AB)}, \end{array}$$

and γ_*ij*_ = 0, for *i, j* = 1, 2.

Theorem 2 proves the strict equivalence between classical conjunction data and the model of conjunction built in ℂ^2^ ⊗ ℂ^2^.

## 3. Unitary transformations and data representation

We now investigate how multiple exemplars can be concretely represented using a single concept state. To do so, we use unitary transformations to identify a basis of the realization space where multiple exemplars can be represented simultaneously. In this new framework, concrete representations are consistent with the cognitive principles of the quantum model of concepts. Namely, a concept exists in a single state for all exemplars, and the measurement of membership of an exemplar depends on the exemplar to be measured rather than on the concept state.

### 3.1. Data representation in the first sector

The following definition and theorem introduce the notion of data representation in the first sector that is consistent with the cognitive principles of the quantum model of concepts in ℂ^3^.

Definition 3. *Let* $\mu ={\left\{\mu_{i}\left(A\right),\mu_{i}\left(B\right),\mu_{i}\left(AB\right)\right\}}_{i=1}^{k}$ *be a set of experimental data, where μ_i_(x) is the semantic estimation of an exemplar p_i_ with respect to concepts* A, B, *and their conjunction* AB. *A representation of μ in* ℂ^3^ *is defined as a pair of unit vectors |A〉, |B〉 ∈* ℂ^3^, *and a collection of orthogonal projectors* ${\text{M}}_{i}:{\mathbb{C}}^{3}\to {\mathbb{C}}^{3}$ *such that conditions* (13–16) *are satisfied for i* = 1, …, *k. We say* $\left(|A\rangle ,\;|B\rangle ,\;{\left\{M_{i}\right\}}_{i=1}^{k}\right)$ *is a representation of μ in* ℂ^3^.

Theorem 3. *Let* $\mu ={\left\{\mu_{i}\left(A\right),\mu_{i}\left(B\right),\mu_{i}\left(AB\right)\right\}}_{i=1}^{k}$ *be a set of experimental data, where μ_i_(x) is the semantic estimation of exemplar p_i_ with respect to concepts* A, B, *and their conjunction* AB. *The set of data μ has a representation in* ℂ^3^ *if and only if for all i* = 1, …, *k*

(53) $$\mu_{i}(AB)\in [{\text{ave}}_{i}(AB)-{\text{dev}}_{i}(AB),{\text{ave}}_{i}(AB)+{\text{dev}}_{i}(AB)].$$

*Proof*. Let |*A*〉 = (1, 0, 0), |*B*〉 = (0, 1, 0), and |*C*〉 = (0, 0, 1) be the canonical basis for ℂ^3^. We prove that, if Equation (53) is satisfied for each *i* = 1, …, *k* then there exists an orthogonal projector **M**_*i*_ such that conditions (13–16) are satisfied for |*A*〉, |*B*〉, and **M**_*i*_.

Since μ_*i*_(*A*), μ_*i*_(*B*) and μ_*i*_(*AB*) satisfy (Equation 53), by Theorem 1 for each *i* ∈ {1, …, *k*} there exist two vectors, 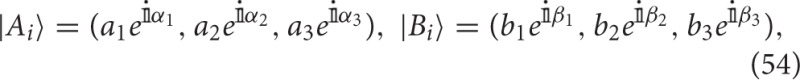 and an orthogonal projector ${\hat{\text{M}}}_{i}$ such that Equations (13–16) are satisfied. Thus, the pair of vectors |*A*_*i*_〉 and |*B*_*i*_〉, as constructed in the proof of Theorem 1, are orthonormal. We set |*C*_*i*_〉 = |*A*_*i*_〉 × |*B*_*i*_〉 so that the set {|*A*_*i*_〉, |*B*_*i*_〉, |*C*_*i*_〉} forms an orthonormal basis for ℂ^3^ for any *i* ∈ {1, …, *k*}. Next, we define the operator U_*i*_ by

(55) $${\text{U}}_{i}=\left(\begin{array}{ccc} \langle A_{i}|A\rangle & \langle A_{i}|B\rangle & \langle A_{i}|C\rangle \\ \langle B_{i}|A\rangle & \langle B_{i}|B\rangle & \langle B_{i}|C\rangle \\ \langle C_{i}|A\rangle & \langle C_{i}|B\rangle & \langle C_{i}|C\rangle \end{array}\right).$$

U_*i*_ is a unitary matrix whose action induces a change from the basis {|*A*_*i*_〉, |*B*_*i*_〉, |*C*_*i*_〉} to the basis {|*A*〉, |*B*〉, |*C*〉}. Note that U_*i*_|*A*_*i*_〉 = |*A*〉, U_*i*_|*B*_*i*_〉 = |*B*〉, and U_*i*_|*C*_*i*_〉 = |*C*〉.

We can also use the operator U_*i*_ to represent ${\hat{\text{M}}}_{i}$ in the canonical basis {|*A*〉, |*B*〉, |*C*〉} as follows:

(56) $${\text{M}}_{i}={\text{U}}_{i}{\hat{\text{M}}}_{i}{\text{U}}_{i}^{-1}.$$

We use the fact that $\text{I}={\text{U}}_{i}^{-1}{\text{U}}_{i}={\text{U}}_{i}{\text{U}}_{i}^{-1}$ to show that the remaining conditions are satisfied. That is, for each *i* = 1, …, *k*,

(57) $$\begin{array}{l} \mu_{i}(A)=\langle A_{i}|{\hat{\text{M}}}_{i}|A_{i}\rangle =\langle A_{i}{\text{U}}_{i}^{-1}|{\text{U}}_{i}{\hat{\text{M}}}_{i}{\text{U}}_{i}^{-1}|{\text{U}}_{i}A_{i}\rangle =\langle A|{\text{M}}_{i}|A\rangle ,\\ \mu_{i}(B)=\langle B_{i}|{\hat{\text{M}}}_{i}|B_{i}\rangle =\langle B_{i}{\text{U}}_{i}^{-1}|{\text{U}}_{i}{\hat{\text{M}}}_{i}{\text{U}}_{i}^{-1}|{\text{U}}_{i}B_{i}\rangle =\langle B|{\text{M}}_{i}|B\rangle , \end{array}$$

and

(58) $$\begin{array}{l} \mu_{i}(AB)=\frac{1}{2}(\mu (A)+\mu (B))+ℜ(\langle A_{i}|{\hat{M}}_{i}|B_{i}\rangle )\\ \;=\frac{1}{2}(\mu (A)+\mu (B))+ℜ(\langle A_{i}{\text{U}}_{i}^{-1}|{\text{U}}_{i}{\hat{M}}_{i}{\text{U}}_{i}^{-1}|{\text{U}}_{i}A_{i}\rangle )\\ \;=\frac{1}{2}(\mu (A)+\mu (B))+ℜ(\langle A|M_{i}|B\rangle ). \end{array}$$

Theorem 3 provides a data representation in terms of a single pair of vectors |*A*〉 and |*B*〉, and a set of projectors **M**_*i*_, for *i* = 1, …, *k*, corresponding to the membership operator for each exemplar. Since the unitary transformations preserve the inner product between vectors and operators, the values of the membership estimations μ_*i*_(*A*), μ_*i*_(*B*), and μ_*i*_(*AB*) are preserved.

Consider for example the exemplars *p* =“filing cabinet” and *q* =“heated waterbed” mentioned in Section 1.3. These can now be represented by the states |*A*〉 = (1, 0, 0), |*B*〉 = (0, 1, 0) and the following measurement operators 
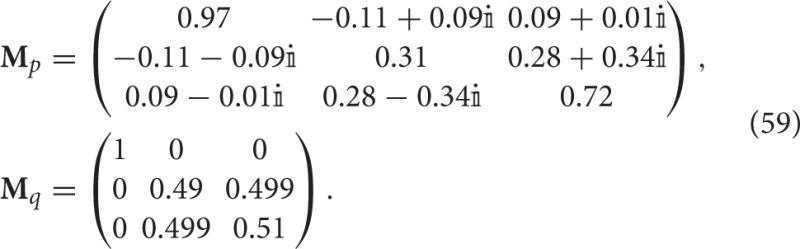
 From a geometric perspective, the operators **M**_*p*_ and **M**_*q*_ correspond to rotations of the one-dimensional projector **M**(*x, y, z*) → (*x*, 0, 0) in ℂ^3^.

### 3.2. Data representation in the second sector

We now apply unitary transformations in the concrete representations of the tensor product model in ℂ^*n*^ ⊗ ℂ^*n*^. We first define different types of representations for multiple exemplars, and then provide explicit representation theorems for the cases *n* = 2 and 3.

Definition 4. *A zero-type representation of* $\mu_{i=1}^{k}$ *on the tensor product space ℂ^n^ ⊗* ℂ^*n*^ *is a unit vector |C〉 ∈* ℂ^*n*^ ⊗ ℂ^*n*^, *and a collection of orthogonal projectors* ${\left\{{\text{M}}_{i}^{A},{\text{M}}_{i}^{B}\right\}}_{i=1}^{k}$ *from* ℂ^*n*^ ⊗ ℂ^*n*^ *to* ℂ^*n*^ ⊗ ℂ^*n*^, *such that conditions* (47)–(49) *are satisfied with* ${\text{M}}_{i}^{\wedge }={\text{M}}_{i}^{A}{\text{M}}_{i}^{B}$, *for i* = 1, …, *k. We say* $\left(|C\rangle ,\;|{\left\{M_{i}^{A},M_{i}^{B}\right\}}_{i=1}^{k}\right)$ *is a zero-type representation of* $\mu_{i=1}^{k}$ *in* ℂ^*n*^ ⊗ ℂ^*n*^.

The zero-type representation is, mathematically speaking, the most general representation in the tensor product model that is consistent with the modeling principles of quantum cognition because it assumes a single concept state |*C*〉, and a collection of measurements that represent the membership weight estimations. However, this representation cannot be appropriately interpreted because ${\text{M}}_{i}^{A}$ and ${\text{M}}_{i}^{B}$ can be entangled measurements, for *i* = 1, …, *k*.

A more reasonable representation of data assumes that ${\text{M}}_{i}^{A}={\text{M}}_{i}⊗𝟙$, and ${\text{M}}_{i}^{B}=𝟙⊗{\text{M}}_{i}$, for *i* = 1, …, *k*. Therefore, these operators are not entangled because they act on different sides of ℂ^3^ ⊗ ℂ^3^.

Definition 5. *A first-type representation of* $\mu_{i=1}^{k}$ *on the tensor product space* ℂ^*n*^ ⊗ ℂ^*n*^ *is a unit vector |C〉 ∈* ℂ^*n*^ ⊗ ℂ^*n*^, *and a collection of orthogonal projectors* **M**_*i*_ *from* ℂ^*n*^ *to* ℂ^*n*^, *for i* = 1, …, *k, such that* (|*C*〉, {**M**_*i*_ ⊗ 𝟙, 𝟙 ⊗ **M**_*i*_}^*k*^_*i* = 1_) *is a zero-type representation of* $\mu_{i=1}^{k}$ *in* ℂ^*n*^ ⊗ ℂ^*n*^.

The first-type representation is a direct extension of the representation of individual exemplars in Definition 2, and thus it is interpreted according to such representation: The state |*C*〉 describes the situation of having two concepts and their combination, and **M**_*i*_ represents the semantic estimation of exemplar *p*_*i*_, *i* = 1, …, *k*.

The zero- and first-type representations require different conditions to model a collection of exemplars for a pair of concepts and their conjunction. While the first-type corresponds to the natural way to represent a pair of systems in quantum physics, and thus is the natural way to define a representation in the tensor product model for concepts, the zero-type provides a more general way to build concrete representations because it does not impose a product structure on the concept state or on the membership operators for the exemplars.

In fact, from Definitions 4–5 it is trivial to deduce that a first-type representation is also a zero-type representation.

The following theorem characterizes the cases when a set of data has a zero-type representation in ℂ^2^ ⊗ ℂ^2^.

Theorem 4. *The set of data* $\mu_{i=1}^{k}$ *has a zero-type representation in* ℂ^2^ ⊗ ℂ^2^ *if and only if μ_i_ is classical conjunction data for i* = 1, …, *k*.

Proof. For each *i* = 1, …, *k*, we use the construction in the proof of Theorem 2 to obtain a tensor $|\tilde{C_{i}}\rangle$ and a one-dimensional projector $\tilde{\text{M}}$ such that ${\tilde{\text{M}}}_{i}^{A}=\tilde{\text{M}}⊗𝟙$, ${\tilde{\text{M}}}_{i}^{B}=𝟙⊗\tilde{\text{M}}$, and ${\tilde{\text{M}}}_{i}^{\wedge }=\tilde{\text{M}}⊗\tilde{\text{M}}$. This gives the tensor product representation of μ_*i*_. Next, we use unitary transformations to change this representation so that $|\tilde{C_{i}}\rangle$ is a vector in the canonical basis of ℂ^2^ ⊗ ℂ^2^. To facilitate the notation, we will make use of the isomorphism 𝕀 between ℂ^2^ ⊗ ℂ^2^ and ℂ^4^. Let

(60) $$\begin{array}{l} (1,0,0,0)=|e_{1}\rangle ,\\ (0,1,0,0)=|e_{2}\rangle ,\\ (0,0,1,0)=|e_{3}\rangle ,\\ (0,0,0,1)=|e_{4}\rangle . \end{array}$$

We define

(61) $$\begin{array}{l} \text{𝕀}(|1\rangle ⊗|1\rangle )=|e_{1}\rangle ,\\ \text{𝕀}(|1\rangle ⊗|2\rangle )=|e_{2}\rangle ,\\ \text{𝕀}(|2\rangle ⊗|1\rangle )=|e_{3}\rangle ,\\ \text{𝕀}(|2\rangle ⊗|2\rangle )=|e_{4}\rangle . \end{array}$$

The isomorphism 𝕀 allows us to represent $|\tilde{C_{i}}\rangle$ by a vector |*C*_*i*_〉 in ℂ^4^.

We can prove the theorem by building a unitary transformation that takes |*C*_*i*_〉 to one of the canonical basis vectors of ℂ^4^, and use this transformation to represent the operators ${\tilde{\text{M}}}^{A},\;{\tilde{\text{M}}}^{B},$ and ${\tilde{\text{M}}}^{\wedge }$ by the operators **M**^*A*^,**M**^*B*^, and **M**^∧^ in ℂ^4^. Next, we apply the the inverse isomorphism 𝕀^−1^ to map these new representations to ℂ^2^ ⊗ ℂ^2^.

Let |*D*_*i*_〉, |*E*_*i*_〉, |*F*_*i*_〉 be three vectors in ℂ^4^ such that

(62) $$\begin{array}{l} \langle D_{i}|D_{i}\rangle =\langle E_{i}|E_{i}\rangle =\langle F_{i}|F_{i}\rangle =1,\\ \langle C_{i}|D_{i}\rangle =\langle C_{i}|E_{i}\rangle =\langle C_{i}|F_{i}\rangle =0,\\ \langle D_{i}|E_{i}\rangle =\langle D_{i}|F_{i}\rangle =\langle E_{i}|F_{i}\rangle =0. \end{array}$$

The vectors |*C*_*i*_〉, |*D*_*i*_〉, |*E*_*i*_〉, and |*F*_*i*_〉 form an orthonormal basis for ℂ^4^.

Let

(63) $${\text{U}}_{i}=\left(\begin{array}{cccc} \langle C_{i}|e_{1}\rangle & \langle C_{i}|e_{2}\rangle & \langle C_{i}|e_{3}\rangle & \langle C_{i}|e_{4}\rangle \\ \langle D_{i}|e_{1}\rangle & \langle D_{i}|e_{2}\rangle & \langle D_{i}|e_{3}\rangle & \langle D_{i}|e_{4}\rangle \\ \langle E_{i}|e_{1}\rangle & \langle E_{i}|e_{2}\rangle & \langle E_{i}|e_{3}\rangle & \langle E_{i}|e_{4}\rangle \\ \langle F_{i}|e_{1}\rangle & \langle F_{i}|e_{2}\rangle & \langle F_{i}|e_{3}\rangle & \langle F_{i}|e_{4}\rangle \end{array}\right).$$

Note that U_*i*_ is a unitary matrix whose action induces a change from the basis {|*C*_*i*_〉, |*D*_*i*_〉, |*E*_*i*_〉, |*F*_*i*_〉} to the basis $\{|e_{j}\rangle {\}}_{j=1}^{4}$. In fact,

$${\text{U}}_{i}|C_{i}\rangle =|e_{1}\rangle ,\;{\text{U}}_{i}|D_{i}\rangle =|e_{2}\rangle ,\;{\text{U}}_{i}|E_{i}\rangle =|e_{3}\rangle {\text{, and U}}_{i}|F_{i}\rangle =|e_{4}\rangle .$$

The operator U_*i*_ can now be used to change the basis in which ${\text{M}}_{i}^{A},{\text{M}}_{i}^{B}$, and ${\text{M}}_{i}^{\wedge }$ are represented, to the basis $\{|e_{j}\rangle {\}}_{j=1}^{4}$:

(64) $$\begin{array}{l} {\bar{M}}_{i}^{A}={\text{U}}_{i}M_{i}^{A}{\text{U}}_{i}^{-1},\\ {\bar{M}}_{i}^{B}={\text{U}}_{i}M_{i}^{B}{\text{U}}_{i}^{-1},\\ {\bar{M}}_{i}^{\wedge }={\text{U}}_{i}M_{i}^{\wedge }{\text{U}}_{i}^{-1}. \end{array}$$

Since 𝟙 = U^−1^_*i*_U_*i*_ = U_*i*_U^−1^_*i*_, we obtain

(65) $$\begin{array}{l} \mu_{i}(A)=\langle C_{i}|M_{i}^{A}|C_{i}\rangle =\langle C_{i}{\text{U}}_{i}^{-1}|{\text{U}}_{i}M_{i}^{A}{\text{U}}_{i}^{-1}|{\text{U}}_{i}C_{i}\rangle =\langle e_{1}|{\bar{M}}_{i}^{A}|e_{1}\rangle ,\\ \mu_{i}(B)=\langle C_{i}|M_{i}^{B}|C_{i}\rangle =\langle C_{i}{\text{U}}_{i}^{-1}|{\text{U}}_{i}M_{i}^{B}{\text{U}}_{i}^{-1}|{\text{U}}_{i}C_{i}\rangle =\langle e_{1}|{\bar{M}}_{i}^{B}|e_{1}\rangle ,\\ \mu_{i}(AB)=\langle C_{i}|M_{i}^{\wedge }|C_{i}\rangle =\langle C_{i}{\text{U}}_{i}^{-1}|{\text{U}}_{i}M_{i}^{\wedge }{\text{U}}_{i}^{-1}|{\text{U}}_{i}C_{i}\rangle =\langle e_{1}|{\bar{M}}_{i}^{\wedge }|e_{1}\rangle . \end{array}$$

We then use the inverse isomorphism 𝕀^−1^ to obtain a zero-type representation in ℂ^2^ ⊗ ℂ^2^:

(66) $$\begin{array}{l} |C\rangle ={\text{𝕀}}^{-}1(|e_{1}\rangle )=|1\rangle ⊗|1\rangle ,\\ {\tilde{M}}_{i}^{A}={\text{𝕀}}^{-1}{\bar{M}}_{i}^{A}\text{𝕀},\\ {\tilde{M}}_{i}^{B}={\text{𝕀}}^{-1}{\bar{M}}_{i}^{B}\text{𝕀},\\ {\tilde{M}}_{i}^{\wedge }={\text{𝕀}}^{-1}{\bar{M}}_{i}^{\wedge }\text{𝕀}. \end{array}$$

We have constructed a zero-type representation $\left(|1\rangle \;⊗\;|1\rangle {\left\{M_{i}^{A},M_{i}^{B}\right\}}_{i=1}^{k}\right)$ from a collection of representations (|*C*_*i*_〉, **M**) for the exemplars *p*_*i*_ with **M**(*x, y*) → (*x*, 0) obtained from Theorem 2.

In the construction of Theorem 4, note that when Equation (66) entails operators ${\text{M}}_{i}^{A}$ and ${\text{M}}_{i}^{B}$ that are of the form ${\text{M}}_{A}^{i}={\overset{ˇ}{\text{M}}}_{i}⊗𝟙$ and ${\text{M}}_{B}^{i}=𝟙⊗{\hat{\text{M}}}_{i}$, then the representation is also of the first-type.

Stating the necessary and sufficient conditions required for a set of data to have first-type representation is out of the scope of this paper. However, we now introduce another type of representation that is mathematically simpler, and can be used to obtain sufficient conditions for a first-type representation.

Definition 6. *A second-type representation of* $\mu_{i=1}^{k}$ *on the tensor product space* ℂ^*n*^ ⊗ ℂ^*n*^ *is a pair of unit vectors |A〉, B〉 ∈* ℂ^*n*^, *and a collection of orthogonal projectors* **M**_*i*_ *from* ℂ^*n*^ *to* ℂ^*n*^, *for i* = 1, …, *k, such that* (|*A*〉⊗|*B*〉, {**M**_*i*_⊗𝟙, 𝟙⊗**M**_*i*_}^*k*^_*i* = 1_) *is a zero-type representation of* $\mu_{i=1}^{k}$ *in* ℂ^*n*^ ⊗ ℂ^*n*^.

The second-type is a mathematical simplification of the first-type representation that assumes |*C*〉 to be a product state.

Lemma 1. *The set of data* $\mu_{i=1}^{k}$ *has a second-type representation in* ℂ^2^ ⊗ ℂ^2^ *if and only if for each i* = 1, …, *k there exist* $|A_{i}\rangle ,|B_{i}\rangle ,{\overset{ˇ}{M}}_{i}^{A}$, and ${\overset{ˇ}{\text{M}}}_{i}^{B}$ *such that Equations (45–47) are satisfied*.

Proof. Let ${\text{U}}_{i}\left(A\right),{\text{U}}_{i}\left(B\right):{\mathbb{C}}^{2}\to {\mathbb{C}}^{2}$ be the unitary transformations that map |*A*_*i*_〉 to |1〉 and |*B*_*i*_〉 to |1〉 respectively, for *i* = 1, …, *k*. Then, it is straightforward to show that $\left(|1\rangle ⊗|1\rangle ,{\left\{M_{i}^{A}⊗1,1⊗M_{i}^{B}\right\}}_{i=1}^{k}\right)$ is a second-type representation of $\mu_{i=1}^{k}$ with

(67) $$\begin{array}{l} M_{i}^{A}=U_{i}{\left(A\right)}^{-1}{\overset{ˇ}{M}}_{i}^{A}U_{i}\left(A\right),\\ M_{i}^{B}=U_{i}{\left(B\right)}^{-1}{\overset{ˇ}{M}}_{i}^{B}U_{i}\left(B\right). \end{array}$$

Theorem 4 and Lemma 1 characterize the sets of data that have a zero- and second-type representations. Since the first-type representation is less general than the zero-type representation, but more general than the second-type representation, these results can be applied to obtain an upper and lower bound for the number of exemplars that have a first-type representation in a given set of data.

Note that Theorem 4 is built in ℂ^2^ ⊗ ℂ^2^. We now extend our results to ℂ^3^ ⊗ ℂ^3^ so they become compatible with the representation analysis developed in Section 3.1 for a Hilbert space model in ℂ^3^. The next corollary extends the proof of Theorem 4 to the space ℂ^3^ ⊗ ℂ^3^.

Corollary 2. *If the set of data* $\mu_{i=1}^{k}$ *has a zero-type representation in* ℂ^2^ ⊗ ℂ^2^, *then* $\mu_{i=1}^{k}$ *has a zero-type representation in* ℂ^3^ ⊗ ℂ^3^.

*Proof*. Let $\left(|C\rangle ,{\left\{M_{i}^{A},M_{i}^{B}\right\}}_{i=1}^{k}\right)$ be a zero-type representation of $\mu_{i=1}^{k}$ in ℂ^2^ ⊗ ℂ^2^. We can create a vector

(68) $$|C^{*}\rangle =\sum_{i,j=1}^{3}c_{ij}^{*}|i\rangle \;⊗|j\rangle$$

such that it is the trivial embedding of

(69) $$|C\rangle =\sum_{i,j=1}^{2}c_{ij}|i\rangle ⊗|j\rangle$$

in ℂ^3^ ⊗ ℂ^3^ by choosing

(70) $$c_{ij}^{*}=\{\begin{array}{cc} c_{ij} & i,j\in \{1,2\}\\ 0 & \text{ else}. \end{array}$$

Similarly, we can also create operators ${\text{M}}_{i}^{A^{*}}$ and ${\text{M}}_{i}^{B^{*}}$ by using the trivial embedding in such a way that the actions of the operators ${\text{M}}_{i}^{A}$ and ${\text{M}}_{i}^{B}$ on ℂ^2^ ⊗ ℂ^2^ are preserved. This completes the proof.

Since second-type representations are also first- and zero-type representations, we can apply Corollary 3.2 to obtain a first- and second-type representation in ℂ^3^ ⊗ ℂ^3^.

## 4. A conjecture about compatibility of exemplars

In quantum theory, measurement operators can be incompatible. That is, when we consider two different observables, the result of their sequential application can depend on the order in which they are applied. The fact that quantum measurements can be incompatible is related to fundamental differences between the quantum and classical realms, such as the observer phenomena, and the Heisenberg uncertainty principle (Heisenberg, 1927; Isham, 2001).

Definition 7. *Given two operators* **M**_1_ and **M**_2_ *represented in the same basis. We say that* **M**_1_ *and* **M**_2_ *represent compatible observables if and only if the commutator operator*

(71) $$\text{[}M_{1}\text{,}M_{2}\text{] = }M_{1}M_{2}-M_{2}M_{1}\text{ = 0.}$$

*Otherwise, the operators represent incompatible observables*.

In terms of cognitive phenomena, sequential measurements could be interpreted as consecutive cognitive actions where the previous action serves as a context for the next action (Busemeyer and Wang, 2007; Wang and Busemeyer, 2013). Since in our concrete representations membership operators are represented in the same basis for all exemplars *p*_*i*_ = 1, …, *k*, it is now possible to test whether or not these measurement operators commute. If we find exemplars whose operators are non-commutative, then we can conjecture the existence of a fundamental limit to the precision with which the membership of these exemplars can be known simultaneously.

Note that we would expect that classical probabilistic models should be compatible, and because the classical probabilistic model and the tensor product model are equivalent, tensor product operators obtained from the data should also be compatible for the vector representing the conceptual situation. However, Hilbert space models could exhibit incompatible measurements for certain data on concept combination, as the Hilbert space model represents non-classical measurements.

We introduce the following definitions to characterize the compatibility of exemplars in ℂ^3^ and in ℂ^3^ ⊗ ℂ^3^:

Definition 8. *Let* |*A*〉 = (1, 0, 0), |*B*〉 = (0, 1, 0), *and* {**M**_1_, **M**_2_} *be a representation in* ℂ^3^ *of* ${\left\{\left(\mu_{i}\left(A\right),\mu_{i}\left(B\right),\mu_{i}\left(AB\right)\right)\right\}}_{i=1}^{2}$, *and set*

(72) $$\begin{array}{l} c_{A}=\langle A|[M_{1},M_{2}]|A\rangle ,\\ c_{B}=\langle B|M_{1},M_{2}]|B\rangle ,\\ c_{AB}=\frac{1}{2}(\langle A|+\langle B|)[M_{1},M_{2}](|A\rangle \;+\;|B\rangle ). \end{array}$$

*We say p*_1_ *and p*_2_ *are compatible with respect to the concepts* A, B, and AB *if and only if c_A_* = 0, *c*_*B*_ = 0, *and c_AB_* = 0, *respectively*.

For simplicity, we will study compatibility for zero-type representations in ℂ^3^ ⊗ ℂ^3^.

Definition 9. *Let* |*C*〉 = (1, 0, 0) ⊗ (1, 0, 0), $\left\{{\text{M}}_{1}^{A},{\text{M}}_{1}^{B},{\text{M}}_{1}^{\wedge }\right\}$, *and* $\left\{{\text{M}}_{2}^{A},{\text{M}}_{2}^{B},{\text{M}}_{2}^{\wedge }\right\}$ be a zero-type representation of data in ℂ^3^ ⊗ ℂ^3^ *of* ${\left\{\left(\mu_{i}\left(A\right),\mu_{i}\left(B\right),\mu_{i}\left(AB\right)\right)\right\}}_{i=1}^{2}$, *and set*

(73) $$\begin{array}{l} {c^{\prime }}_{A}=\langle C|[M_{1}^{A},M_{2}^{A}]|C\rangle ,\\ {c^{\prime }}_{B}=\langle C|[M_{1}^{B},M_{2}^{B}]|C\rangle ,\\ {c^{\prime }}_{AB}=\langle C|[M_{1}^{\wedge },M_{2}^{\wedge }]|B\rangle ). \end{array}$$

*We say p*_1_ *and p*_2_ *are compatible with respect to concepts* A, B, *and* AB *if and only if* $c_{A}^{\prime }=0$, $c_{B}^{\prime }=0$, and $c_{AB}^{\prime }=0$, *respectively*.

We have verified the compatibility of exemplars for each conceptual combination that can be modeled in ℂ^3^ and in ℂ^3^ ⊗ ℂ^3^ using the data in Hampton (1988a,b). The results support our predictions. We have found that the tensor product model always leads to compatible measurements, and that the Hilbert space model leads to incompatible measurements in most cases.

For example, consider the concepts A = “*Machine”* and B = “*Vehicle*,” and the exemplars *p*_5_ = “sailboat” and *p*_12_ = “skateboard.” For the case of conceptual conjunction, we have

(74) $$\begin{array}{l} \mu_{5}(A)=0.56,\mu_{5}(B)=0.8,\mu_{5}(AB)=0.42,\text{ and}\\ \mu_{12}(A)=0.28,\mu_{12}(B)=0.84,\mu_{12}(AB)=0.34. \end{array}$$

Note that exemplar *p*_5_ satisfies the conditions of Theorems 1 and 2. Thus, it can be represented in both ℂ^3^ and in ℂ^3^ ⊗ ℂ^3^. However, the exemplar *p*_12_ is singly overextended. Therefore, we can only represent the two exemplars simultaneously in ℂ^3^.

When we apply Theorems 1 and 3, and Definition 8, on these data sets, we obtain 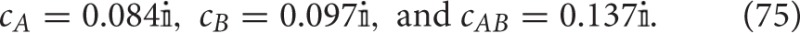 Thus, exemplars *p*_5_ and *p*_12_ are incompatible. Moreover, note that the incompatibility is larger for the conjunction of the concepts than for each of the former concepts.

As a second example, consider the concepts A = “*Building*,” and B = “*Dwelling*,” and the exemplars *p*_2_ = “cave,” and *p*_10_ = “synagogue,” whose memberships are given by

(76) $$\begin{array}{l} \mu_{2}(A)=0.28,\;\mu_{2}(B)=0.85,\;\mu_{2}(AB)=0.28,\text{ and}\\ \mu_{10}(A)=0.93,\mu_{10}(B)=0.49,\mu_{10}(AB)=0.45. \end{array}$$

Both exemplars satisfy the conditions of Theorem 2. Applying Theorems 2 and 4, and Definition 9, we obtain

$$c{\text{'}}_{A}\text{ = }c{\text{'}}_{B}\text{ = }c{\text{'}}_{AB}\text{ = 0.}$$

This is consistent with our expectations because the representation in the second sector ℂ^3^ ⊗ ℂ^3^ correspond to classical (and thus compatible) measurements.

Since our data was collected presenting the exemplars in only one specific order (Hampton, 1988b), these computations demonstrate that we can predict order effects by determining the exemplars that are incompatible. The results presented here are, however, speculative since there is no experimental data where order effects have been recorded that could be used to contrast our computations. While our data set does not allow us to make a strong claim, we conjecture that order effects are predictable, and suggest that the concrete representations proposed in this paper could be used to develop Heisenberg-like uncertainty relations in the context of conceptual combinations.

## 5. Conclusion and future work

In this paper, we have made some advances on the representational aspects of the quantum model for concept combinations. First, we proved that the first and second sectors of the two-sector Fock space model of concept conjunctions can be concretely represented in ℂ^3^ and ℂ^3^ ⊗ ℂ^3^, respectively. Next, we introduced unitary transformations to provide concrete representations that are consistent with the cognitive principles of the quantum model of concepts, and used these concrete representations to study the question of measurement compatibility.

The representations introduced here could be an important tool for future applications. First, since they are consistent with the cognitive principles of the quantum model of concepts, the model could easily be introduced to a wider audience, and extended to produce concrete representations in the two-sector Fock space model. Second, they can be adopted as a representational standard for different groups who seek to develop their own computational implementations of the model. Third, the fact that all the measurements are represented in a single basis constitutes a tremendous mathematical advantage for studying the probabilistic structure of concepts.

The evidence obtained in the application of our representations to the issue of exemplar compatibility is consistent with the assumptions of the model. Since the second sector entails logical reasoning, measurements in the tensor product model should be compatible. However, incompatible measurements are likely to be found in the Hilbert space model, since the first sector is associated with non-logical or intuitive reasoning. Moreover, this line of enquiry invites us to explore possible relations between the projector operator structure and the meaning of the exemplar.

In summary, the introduction of unitary transformations and the subsequent application to develop concrete representations of concepts and their combinations seems to be a promising line of research that has the potential to expand both theoretical and applied research in quantum cognition.

## Funding

This research has been funded by an internal grant from the I.K Barber school of Arts and Sciences at UBC Okanagan.

### Conflict of interest statement

The authors declare that the research was conducted in the absence of any commercial or financial relationships that could be construed as a potential conflict of interest.
