# Supplementary material for: Unitary Transformations in the Quantum Model for Conceptual Conjunctions and Its Application to Data Representation
Source: Front Psychol. 2015 Nov 12;6:1734. doi: 10.3389/fpsyg.2015.01734 (PMC4642215; doi:10.3389/fpsyg.2015.01734)
Supplement: Supplementary file 1 [file Table1.PDF]

Supplementary Material: Unitary Transformations in the Quantum Model for Conceptual Combinations and its Application to Data Representation

Tomas Veloz[1,2,3], **Sylvie Desjardins**[1]
*Correspondence:
Author Name: Tomas Veloz
tveloz@gmail.com

1 SUPPLEMENTARY DATA

Supplementary Material should be uploaded separately on submission. Please include any supplementary data, figures and/or tables.

2 SUPPLEMENTARY TABLES AND FIGURES

	$\mathcal{A}_1$	$\mathcal{B}_1$	$\mathcal{A}_1\mathcal{B}_1$	$\mathcal{A}_2$	$\mathcal{B}_2$	$\mathcal{A}_2\mathcal{B}_2$
$\mu(x_1)$	1	0.18	0.35	-	-	-
$\mu(x_2)$	-	-	-	0.5	0.9	0.95

Table 1. Experimental membership μ for exemplars $x_1 =$'coffee table,' and $x_2 =$'tree house.'
