# Supplementary material for: Unitary Transformations in the Quantum Model for Conceptual Conjunctions and Its Application to Data Representation
Source: Front Psychol. 2015 Nov 12;6:1734. doi: 10.3389/fpsyg.2015.01734 (PMC4642215; doi:10.3389/fpsyg.2015.01734)
Supplement: Supplementary file 2 [file DataSheet1.PDF]

# Unitary Transformations in the Quantum Model for Conceptual Conjunctions and its Application to Data Representation

**Tomas Veloz** [1,2,3], **Sylvie Desjardins**[1]

[1] *Department of Mathematics, University of British Columbia - Okanagan, Kelowna, BC, Canada.*
[2] *Center Leo Apostel (CLEA), Vrije Universiteit Brussel (VUB), Krijgskundestraat 33, 1160 Brussels, Belgium.*
[3] *Instituto de Filosofía y Ciencias de la Complejidad - IFICC. Los Alerces 3024, Ñuñoa, Chile.*

Correspondence*:
Tomas Veloz
Department of Mathematics, University of British Columbia - Okanagan, University Way 3333, Kelowna, BC, V1YV17, Canada., tveloz@gmail.com

## 1 APPENDIX: BASICS OF QUANTUM COGNITION MODELING

This section introduces some mathematical elements of standard quantum mechanics, and shows how they can be applied to cognition.

### 1.1 Quantum Modeling in a Hilbert Space

In quantum physics, the state of a quantum entity is described by a complex-valued vector of unit length. Vectors are denoted using the bra-ket notation introduced by Paul Dirac **?**. In Dirac notation, there are two types of vectors: 'bra' vectors denoted by $\langle A|$, and 'ket' vectors denoted by $|A\rangle$. By convention, the state of a quantum entity is described by a 'ket' vector.

DEFINITION 1. *Let $\alpha, \beta \in \mathbb{C}$. Consider the vectors $\langle A|$ and $|B\rangle$. The operation bra-ket defined by the inner product $\langle A|B\rangle$ is*

1. *linear in the ket: $\langle A|(\alpha|B\rangle + \beta|C\rangle) = \alpha\langle A|B\rangle + \beta\langle A|C\rangle$, and*
2. *anti-linear in the bra: $(\alpha\langle A| + \beta\langle B|)|C\rangle = \alpha^*\langle A|C\rangle + \beta^*\langle B|C\rangle$.*

We say that $|A\rangle$ and $|B\rangle$ are orthogonal if and only if $\langle A|B\rangle = 0$. We denote it by $|A\rangle \perp |B\rangle$. Additionally, we say that $\langle A|B\rangle$ is the complex conjugate of $\langle B|A\rangle$. Therefore

$$\langle A|B\rangle = \langle B|A\rangle^*. \tag{1}$$

DEFINITION 2. *The bra-ket operation induces the norm $||\cdot|| = \sqrt{\langle\cdot|\cdot\rangle}$.*

The space of complex-valued vectors representing the possible states of a quantum entity, equipped with the bra-ket operation and its induced norm, is called a Hilbert space denoted by $\mathcal{H}$. The formalism of quantum mechanics is built upon the mathematics of Hilbert spaces **?**.

Measurable quantities of a system, known as observables in quantum physics, are represented by Hermitian operators on the Hilbert space. We focus on a special type of Hermitian operators used to represent quantum measurements with finite outcomes.

DEFINITION 3. *Let* $|A\rangle$, *and* $|B\rangle \in \mathcal{H}$, *and let* $\mathbf{M} : \mathcal{H} \to \mathcal{H}$, *be an operator defined by*

$$|A\rangle \to \mathbf{M}|A\rangle.$$

$\mathbf{M}$ *is an orthogonal projector if and only if it is*

1. *Linear: for* $\alpha, \beta \in \mathbb{C}$ *we have* $\mathbf{M}(\alpha|A\rangle + \beta|B\rangle) = \alpha\mathbf{M}|A\rangle + \beta\mathbf{M}|B\rangle$,
2. *Hermitian:* $\langle A|\mathbf{M}|B\rangle = \langle B|\mathbf{M}|A\rangle$, *and*
3. *Idempotent:* $\mathbf{M} \cdot \mathbf{M} = \mathbf{M}$.

An orthogonal projector $\mathbf{M}$ is self adjoint. This means that for all states $|A\rangle \in \mathcal{H}$,

$$\langle A\mathbf{M}|A\rangle = \langle A|\mathbf{M}A\rangle \tag{2}$$

Hence $\mathbf{M}^* = \mathbf{M}$.

DEFINITION 4. *Let* $|A\rangle$ *be the state of an entity* $\mathcal{A}$, *and* $\mathbf{M}$ *be an orthogonal projector. The probability of an answer 'yes' to the question measured by* $\mathbf{M}$ *is given by*

$$\mu(A) = \langle A|\mathbf{M}|A\rangle. \tag{3}$$

So far, we have considered a state $|A\rangle$. The quantum model for concept combinations defines the state $|AB\rangle$ of a combined concept as a superposition of states

$$|AB\rangle = \frac{1}{\sqrt{2}}(|A\rangle + |B\rangle). \tag{4}$$

When a measurement $\mathbf{M}$ is applied, the probability $\mu(AB)$ of obtaining an outcome 'yes' is given by

$$\begin{aligned}\mu(AB) &= \frac{1}{2}(\langle A| + \langle B|)\mathbf{M}(|A\rangle + |B\rangle), \\ &= \frac{1}{2}(\langle A|\mathbf{M}|A\rangle + \langle B|\mathbf{M}|B\rangle) + \Re(z_1 z_2^*)\langle A|\mathbf{M}|B\rangle, \\ &= \frac{1}{2}(\mu(A) + \mu(B)) + \Re(\langle A|\mathbf{M}|B\rangle),\end{aligned} \tag{5}$$

where $\Re(\langle A|\mathbf{M}|B\rangle)$ denotes the real part of $\langle A|\mathbf{M}|B\rangle$. Hence, the probability of an outcome 'yes' for the observable represented by $\mathbf{M}$ is the weighted sum of the probabilities of the former events ('yes' on state $|A\rangle$ and 'yes' on state $|B\rangle$) together with an interference term **?**.

### 1.2 Quantum Modeling in the tensor product of Hilbert Spaces

Quantum formalism assumes that quantum systems exist in superposed states. If a quantum system is formed by the composition of sub-systems, then each sub-system exists in its own superposed state, but the behaviour for the emerging system may correspond to that of a non-decomposable entity. In particular, when we perform measurements on the sub-systems of a composite quantum system, the results may reveal that the sub-systems do not behave independently, even if the subsystems are separated by a large distance. Technically speaking, when the emergent system is analyzed, it is possible to encounter states that exhibit non-trivial correlations in the outcomes of their measurements. These states, called entangled states, have inspired some of the most important applications of quantum physics **?**.

Consider for example a composite quantum systems $\mathcal{C}$ obtained by composing two separate quantum systems, $\mathcal{C}_1$ and $\mathcal{C}_2$. Formally, the composition of quantum entities correspond to an element in the tensor product space $\mathcal{H} \otimes \mathcal{H}$.

DEFINITION 5. *Let* $\{|A_i\rangle\}$ *form a basis for* $\mathcal{H}$. *Then, a composite vector* $|C\rangle \in \mathcal{H} \otimes \mathcal{H}$ *is given by*

$$|C\rangle = \sum_{i,j}^{n} c_{ij} |A_i\rangle \otimes |A_j\rangle. \tag{6}$$

DEFINITION 6. *Let* $|C\rangle \in \mathcal{H} \otimes \mathcal{H}$. *If* $|C\rangle$ *can be factorized as* $|C\rangle = |C_1\rangle \otimes |C_2\rangle$, *where* $|C_1\rangle \in \mathcal{H} \otimes \mathbf{1}$, *and* $|C_2\rangle \in \mathbf{1} \otimes \mathcal{H}$, *we say the* $|C\rangle$ *is a separable vector. Otherwise,* $|C\rangle$ *is a non-separable vector, representing an entangled state.*

Because separable vectors can be represented as ordered pairs, when a measurement is performed on one of the sub-systems, the collapse of the wave function induced by the measurement occurs only at the measured sub-system. The other sub-system remains in its original superposed state. Non-separable vectors, however, cannot be factorized as a single tensor product. Therefore, when a measurement is performed on a non-separable vector, the collapse of the wave function induced by the measurement will affect both sub-systems.

The idea of applying the tensor product to model concept conjunctions and disjunctions was first proposed in **?**, and has been later applied to other kinds of combination **?**. We can build a simple tensor product model for the membership of an exemplar with respect to concepts $\mathcal{A}$, $\mathcal{B}$, and their combination $\mathcal{AB}$. Namely, we introduce a state $|C\rangle \in \mathcal{H} \otimes \mathcal{H}$ to represent the situation of the two concepts $\mathcal{A}$, $\mathcal{B}$, and their combination $\mathcal{A}B$.

To obtain the membership for the single concepts $\mathcal{A}$ and $\mathcal{B}$ from the state $|C\rangle$, we require that the membership operators $\mathbf{M}^A = \mathbf{M} \otimes \mathbf{1}$ and $\mathbf{M}^B = \mathbf{1} \otimes \mathbf{M}$ measure the memberships $\mu(A)$ and $\mu(B)$ when applied to the vector $|C\rangle$. Therefore, we require

$$\begin{aligned} \langle C|\mathbf{M}^A|C\rangle &= \langle C|\mathbf{M} \otimes \mathbf{1}|C\rangle = \mu(A), \\ \langle C|\mathbf{M}^B|C\rangle &= \langle C|\mathbf{1} \otimes \mathbf{M}|C\rangle = \mu(B). \end{aligned} \tag{7}$$

and

$$\langle C|\mathbf{M}^{\wedge}|C\rangle = \langle C|\mathbf{M} \otimes \mathbf{M}|C\rangle = \mu(AB). \tag{8}$$

A tensor product model for concept conjunction is given by a vector $|C\rangle$ and an operator $\mathrm{M}$ satisfying Eqs. (7) and (8).
